# Differential Proteomic Analysis of the Hippocampus in Rats with Neuropathic Pain to Investigate the Use of Electroacupuncture in Relieving Mechanical Allodynia and Cognitive Decline

**DOI:** 10.1155/2021/5597163

**Published:** 2021-08-04

**Authors:** Degui Gong, Xiangmei Yu, Menghong Jiang, Changzheng Li, Zhifu Wang

**Affiliations:** ^1^Affiliated Rehabilitation Hospital of Fujian University of Traditional Chinese Medicine, Fuzhou, China; ^2^Fujian University of Traditional Chinese Medicine, Fuzhou, China; ^3^Key Laboratory of Orthopedics & Traumatology of Traditional Chinese Medicine and Rehabilitation, Ministry of Education, China

## Abstract

Abnormal changes in hippocampal function and neuroplasticity are involved in neuropathic pain, which induces hyperalgesia and learning and memory deficits. Previous studies from our group have shown that electroacupuncture at Huantiao (GB30) and Yanglingquan (GB34) has an obvious analgesic effect on neuropathic pain. However, the central regulatory mechanism occurring in the hippocampus remains to be investigated. In this study, behavioral and proteomic analyses were performed to identify differentially expressed hippocampal proteins involved in electroacupuncture-induced analgesia. Our results showed both upregulated (TMEM126A, RDH13, and Luc7L) and downregulated proteins (Mettl7A, GGA1 RTKN, RSBN1, and CDKN1B). Further protein verification revealed for the first time that hippocampal TMEM126A plays an important anti-inflammatory role in the treatment of neuralgia by electroacupuncture.

## 1. Introduction

Cognitive impairment is commonly associated with the experience of pain, with approximately 73%–81% of patients with chronic pain suffering from a memory deficit [1, 2]. Recent clinical studies have reported objective cognitive impairments in patients with peripheral neuropathic pain [3, 4]. Several studies have provided evidence suggesting that structural and functional abnormalities in the hippocampus underlie the cognitive deficits associated with neuropathic pain [5–8]. The hippocampus receives complex integrated sensory and cognitive information, including pain signals. The changes of some gene and proteins such as the upregulation of TNF-*α*, IL-1, and MCP-1 in the hippocampus play key roles in the neuropathic pain accompanied with cognitive impairment in rodents [9, 10]. However, the hippocampal proteins and their related signaling pathways involved in neuropathic pain still remain largely elusive.

Electroacupuncture (EA) at acupoints Huantiao (GB30) and Yanglingquan (GB34) is effective in relieving pain sensitization and cognitive decline associated with neuropathic pain in humans and rodents [11–13]. Spinal opioids, serotonin, norepinephrine, amino acids, and glial cells/cytokines are the primary mediators of EA-induced analgesia of neuropathic pain [14]. Recent studies have focused on the role of hippocampal functional changes in the beneficial effect of EA on the pain condition. Regulation of the hippocampal proteins related to amino acid metabolism and activation of the MAPK signaling pathway is involved in the analgesic effect of EA in neuropathic pain [15, 16]. However, few studies to date have focused on the role of the molecular function of the hippocampus in EA-induced analgesia and cognitive improvement.

Proteomics analysis provides a valuable strategy for exploring the pathogenesis of pain mellitus, as well as therapeutic targets in this condition. We hypothesize that some key genes or proteins in the hippocampus play an essential role in the neuropathic pain with EA treatment. Based on the prior work outlined above, this study was aimed at exploring the molecular mechanisms underlying EA-induced analgesia in the hippocampus using proteomic and behavioral analyses.

## 2. Materials and Methods

### 2.1. Animals and Materials

Male Sprague-Dawley (SD) rats (8–10 weeks, 200–220 g) were obtained from the Shanghai Slack Laboratory Animal Co. (Shanghai, China). The rats were housed under stable temperature conditions (22°C) and a 12/12 h light/dark cycle with free access to food and water at the Experimental Animal Center of Fujian University of Chinese Medicine. SD rats were randomly divided into a Sham operation group (Sham), a spared nerve injury model group (SNI), and an electroacupuncture group (EA). Ten animals per group underwent behavioral testing and analysis, and four animals per group were used for Enzyme-Linked Immunosorbent Assay (ELISA) and western blot testing after all behavioral testing. All animal experimental protocols were approved by the Animal Ethics Committee of Fujian University of Traditional Chinese Medicine (No. FJTCM2019-006). All rats were humanely sacrificed according to the care guidelines. The rats were anesthetized and euthanized with more isoflurane to minimize the pain experienced by the animals.

The following reagents and instruments were used: von Frey filament (North Medical, CA, USA); acupuncture needle, 0.30 mm × 25 mm (Suzhou Medical Supplies Factory, China); Tmem126a ELISA kit (Wuhan Boster Biological Technology, China); TMEM126A rabbit pAb (A12823; ABclonal Technology, China); and GAPDH goat pAb (ab9483; Abcam, USA).

### 2.2. Spared Nerve Injury-Induced Neuropathic Pain Model and EA Stimulation

The spared nerve injury of sciatic nerve was performed under anesthesia according to the procedures described previously [17]. In the SNI and EA groups, the right sciatic nerve branches of rats were exposed and performed selective cutting, or ligation. Briefly, the common peroneal and the tibial nerves were separated, tightly ligated with 5-0 silk, and transected distal to the ligation. A 2 mm length of each nerve was removed, while preserving the integrity of the sural nerve. In the Sham operation group, only the sciatic nerve branch was exposed, without performing any nerve severance or ligation. Rats were monitored for any sign of infection or distress after surgery.

Seven days after spared nerve injury surgery, EA group rats were loosely fixed on a wooden stand that permitted free movement of their head and limbs. The acupoints Huantiao (GB30) and Yanglingquan (GB34) on the right side were selected for acupuncture and electrical stimulation. In the rat, GB30 is located at the junction of the lateral third and medial two-thirds of the line connecting the prominence of greater trochanter of the femur with the sacral hiatus, while GB34 is located in the depression anterior and distal to the head of the fibula [18, 19]. EA stimulation was performed for 30 min, once a day for three consecutive weeks. Needles inserted at GB30 and GB34 were connected to a G6805-1A multifunctional EA apparatus (Shanghai Medical Electronic Apparatus Company, Shanghai, China), using a stimulation intensity of 1 mA and a frequency of 2 Hz. The Sham operation group and model group underwent the experimental protocol at the same time each day.

### 2.3. Behavioral Testing

The mechanical paw withdrawal threshold (PWT) was assessed on day 0, 7, 14, 21, and 28 postsurgery after EA stimulation using von Frey filaments. According to the up-down method, von Frey filaments of different intensities (0.6 g, 1.0 g, 1.4 g, 2.0 g, 4.0 g, 6.0 g, 8.0 g, 15.0 g, and 26.0 g) were used to determine the 50% PWT in rats. A quick paw withdrawal or licking of the paw in response to the stimulus was considered to be a positive response. The protocol for assessing the PWT was based on the one described in our previous study [20].

The novel-object recognition test was performed in a Plexiglas box (60 mm × 60 mm × 50 mm) on day 28 postsurgery. After habituation, on day 1, the rats were placed in the box for 20 min without objects being presented. Four hours later, the rats were placed in the box again and allowed to explore objects A and B for 5 min. After a retention interval of 24 h, a cognitive test was performed in the same box by replacing object B with a novel object C. The rats were then allowed to explore the objects freely for 5 min. The ratio of the difference between the time spent exploring the familiar object and that spent exploring the novel object over the total time spent exploring both objects was used to evaluate cognitive function.

### 2.4. Proteomic Detection and Analysis

#### 2.4.1. Protein Extraction and Trypsin Digestion

All methods of proteomic detection and analysis were according to our team's previous research as follows [21].

Proteins were extracted from the hippocampal tissue samples in lysis buffer (8 M urea, 1% protease inhibitor) using a high-intensity ultrasonic processor. Remaining debris was removed by centrifugation at 12,000 × g for 10 min at 4°C. The protein concentration was detected with a Bicinchoninic Acid (BAC) kit.

For trypsin digestion, protein solution was reduced with dithiothreitol for 30 min at 56°C, then incubated with iodoacetamide for 15 min at 37°C in darkness. Secondly, protein samples were diluted with millimolar Triethylammonium bicarbonate (TEAB), until the urea concentration was <2 M. Finally, trypsin was added at a mass ratio (trypsin : protein) of 1 : 50 for the first digestion at 37°C overnight, then at a ratio of 1 : 100 for the second digestion.

#### 2.4.2. Tandem Mass Tag (TMT) Labeling and LC-MS/MS Analysis

After trypsin digestion, peptides were desalted and vacuum-dried with the Strata X C18 SPE column. Peptides were reconstituted in 0.5 M TEAB and marked according to the TMT kit protocols.

Separated peptides were subjected to sodium/iodide symporter sources. Tandem mass spectrometry (MS/MS) was performed using a Q Exactive Plus (Thermo) instrument, coupled online to an ultraperformance liquid chromatography system.

Using an electrospray voltage of 2.0 kV, intact peptides were then detected in the Orbitrap at 70,000 mass resolution. The primary MS scan range was 350–1,600 *m*/*z*. Collected data were processed using a data-dependent scanning program (DDA). Automatic gain control was set at 50,000, with a signal threshold of 5,000 ions/s, maximum time of 200 s, and dynamic exclusion time of the tandem mass scan of 15 s, to avoid repeated scanning of precursor ions.

#### 2.4.3. Database Search

MS/MS data were analyzed using the MaxQuant search engine (v.1.5.2.8), with the following parameters: rat UniProt was first screened; then, a reverse library was added to calculate the false-positive ratio (FPR); trypsin/P was specified as the cleavage enzyme, allowing up to two missed cleavages; the minimum peptide length was 7 amino acid residues; the maximum number of peptide modifications was 5; the mass tolerance for the primary precursor ions search was 20 ppm, while that for the main search was 5 ppm; the mass tolerance for fragment ions was 0.02 Da; the quantitative method was set to TMT-10plex; and the FPR for peptide-spectrum match identification was set to 1%.

#### 2.4.4. Bioinformatic Annotation

Gene Ontology (GO) annotations were derived from the UniProt-GOA database (http://www.ebi.ac.uk/GOA/). InterProScan soft was used to analyze the following GO categories: molecular function, cellular component, and biological process. The KEGG online service tools were used to annotate protein descriptions, which were matched to their corresponding pathways using the KEGG mapper. WoLFPSort (https://wolfpsort.hgc.jp/) was used to investigate subcellular localization. KEGG database pathway enrichment analysis was conducted using the two-tailed Fisher exact test. Pathways were classified according to the KEGG website.

Further cluster analysis of functional enrichment was conducted to explore potential connections and differences in specific functions. First, data for all GO categories were collected after enrichment, sorted according to their *P* values, then filtered to obtain the data for categories with *P* < 0.05. This filtered *P* value matrix was transformed using the function *x* = −log_10_ (*P*). Finally, the *x* values for each functional category were *z*-transformed. Cluster membership was visualized using a heat map.

### 2.5. ELISA Testing

Concentrations of Tmem126a were analyzed using an ELISA kit according to the manufacturer's instructions. Briefly, 100 *μ*l, respectively, of standard and diluted samples in duplicated wells was incubated at room temperature for 2 h. After the wells were washed 5 times with 1× wash solution, 100 *μ*l enzyme conjugate reagent was added to each well and the wells were incubated for 2 h at room temperature. 100 *μ*l substrate solution was then added, and after 30 min incubation, 100 *μ*l of stop solution was added to terminate the reaction. The resultant color was assayed using a microtiter plate reader.

### 2.6. Western Blot Testing

After spared nerve injury surgery, rats (*n* = 4 per group) were sacrificed on day 28 in order to perform analyses of hippocampal proteins. Protein extraction, sodium dodecyl sulfate–polyacrylamide gel electrophoresis (SDS–PAGE), and western blotting analyses were performed. The protein was extracted using homogenization in SDS sample buffer, followed by centrifugation at 12,000 × g for 20 min. The protein concentration of the supernatant was determined using the BCA Protein Assay Kit (Pierce, Rockford, USA), and 60 *μ*g of protein was loaded into each lane of the 10% SDS–PAGE gel. The membrane was blocked overnight using 5% bovine serum albumin in TBS-T. The blot was then probed using the following primary antibodies: rabbit anti-TMEM126A antibody (1 : 500) and goat anti-GAPDH (1 : 1,000) overnight at 4°C. Then, the blot was incubated with HRP-anti-rabbit/goat (1 : 2,000; Santa Cruz, CA, USA) antibody for 1 h at room temperature. The blots were incubated in ECL (Pierce) solution for 3 min and then exposed onto Kodak X-OMAT AR Film (Eastman Kodak, Rochester, USA) for 1.5 min. Densitometric analysis of the TMEM126A bands was performed using Syngene software (Gene Gnome, Syngene, MD, USA).

### 2.7. Statistical Analysis

All data are presented as the mean ± standard deviation (SD). The three experimental groups were analyzed using the one- and two-way Analysis of Variance (ANOVA) and post hoc Tukey tests in SPSS 21.0 (SPSS, Armonk, NY, USA). Graphs were generated using GraphPad Prism 7.0 software.

## 3. Results

### 3.1. EA Abolishes Mechanical Pain Sensitivity and Memory Deficits Induced by Neuropathic Pain

**Figure 1:**
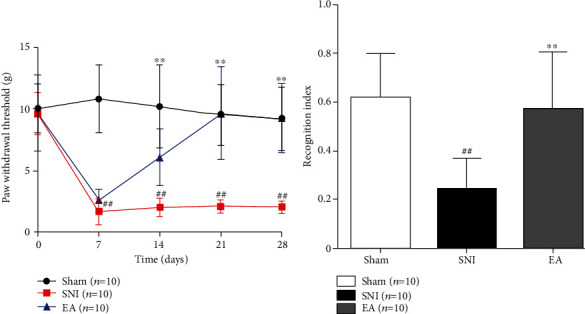
(a) Paw withdrawal in response to mechanical pain varies among the Sham, SNI, and EA groups (two-way repeated ANOVA, *F* = 205.036, *P* < 0.001; post-hoc Tukey test, Sham compared with SNI, ^##^*P* < 0.001; EA compared with SNI, ^∗∗^*P* < 0.001). (b) Significant changes of recognition index detected with new object recognition behavior test in different groups (one way ANOVA, *F* = 12.433, *P* < 0.001; post hoc Tukey test, Sham compared with SNI, ^##^*P* < 0.001; EA compared with SNI, ^∗∗^*P* < 0.001).

As shown in Figure 1(a), compared with the SNI model group, the EA group showed a significantly increased mechanical pain threshold on days 7, 14, and 21 of neuropathic pain (*P* < 0.001). On day 28 of spared nerve injury, as shown in Figure 1(b), the novel object recognition index significantly decreased in the SNI rats (*P* < 0.001). Compared with the SNI model group, the novel object recognition index was significantly increased in the EA group (*P* < 0.001).

### 3.2. Hippocampal Proteomic Changes in SNI Rats Treated with EA

**Figure 2:**
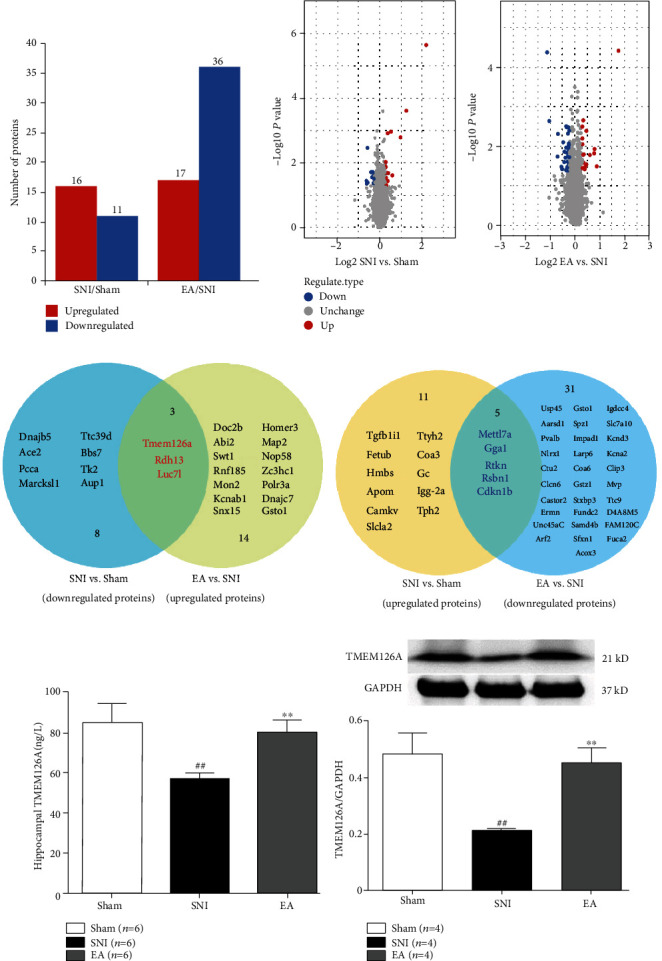
Quantification of differentially expressed proteins in the three groups. (a) Number of differentially expressed protein using a threshold of 1.2-fold (EA group vs. SNI group; SNI group vs. Sham group). (b) Volcanic scatter plot with a threshold of 1.2-fold. (c) Number of EA-regulated SNI-induced differential proteins shown in the Venn diagram using a threshold of 1.2-fold. (d) Hippocampal TMEM126A expression detected with ELISA in the different groups (one-way ANOVA, *F* = 33.049, *P* < 0.001; post hoc Tukey test, Sham group vs. SNI group, ^##^*P* < 0.001; EA group vs. SNI group, ^∗∗^*P* < 0.001; *n* = 6 per group). (e) Hippocampal TMEM126A expression detected via western blot in the different groups (one-way ANOVA, *F* = 31.932, *P* < 0.001; post hoc Tukey test, Sham group vs. SNI group, ^##^*P* < 0.001; EA group vs. SNI group, ^∗∗^*P* < 0.001; *n* = 4 per group).

Compared with the Sham operation group, 16 proteins were upregulated and 11 proteins were downregulated in the hippocampus of the SNI model group. Compared with the SNI model group, 17 proteins were upregulated and 36 proteins were downregulated in the EA group (Figures 2(a) – 2(c)).

Among the 27 regulatory proteins induced by spared nerve injury, the most significantly upregulated or downregulated proteins, respectively, were transmembrane protein 126A (TMEM126A) and excitatory amino acid transporter 2 (SLC1A2/EAAT2), which have mainly been associated with persistent pain, cognitive impairment, and immune-inflammatory regulation (Table 1). ELISA and western blot results showed that after continuous EA, TMEM126A expression was significantly decreased in the SNI group compared with the Sham group, while TMEM126A expression was significantly upregulated, consistent with the proteomic results (Figures 2(d) and 2(e)).

Further proteomic detection and analysis showed that spared nerve injury-induced downregulated proteins such as TMEM126A, RDH13, and Luc71 were upregulated in the EA group. In addition, spared nerve injury-induced upregulated proteins such as MettL7A, GGA1, RTKN, rsBN1, and CDKN1B were significantly downregulated after EA treatment (Table 2).

### 3.3. Analysis of Biological Process, Cell Component, and Molecular Function in SNI and EA Group Rats

**Figure 3:**
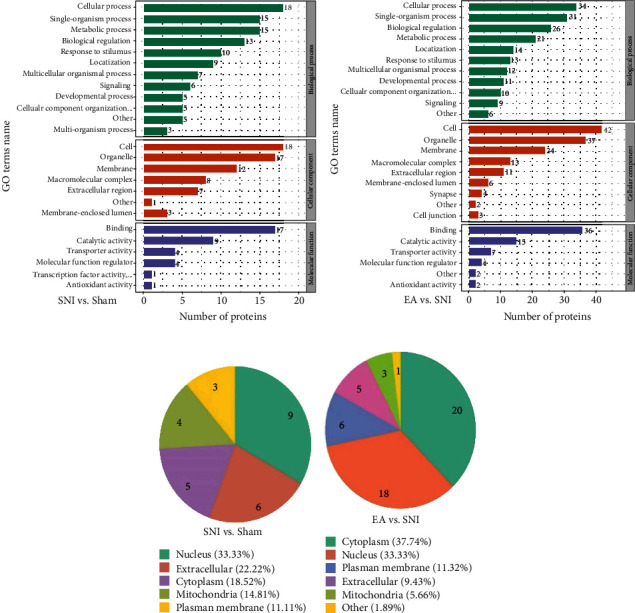
GO annotation analysis showed that the potential biomarkers identified in this study are mainly involved in SNI pathology and EA treatment: (a, b) GO annotation summarizing the numbers of SNI- and EA-regulated proteins with specific molecular function, cellular component, and biological process categories; (c, d) subcellular structural localization analysis of SNI- and EA-associated proteins.

In terms of biological process analysis, the top four regulated processes, respectively, were cellular process, single-organism process, metabolic process, and biological regulation. For the cell component analysis, the most common proteins were mainly associated with the cell and organelle components. The molecular function analysis suggested that the most common molecular functions were binding and catalytic activity (Figures 3(a) and 3(b)).

According to the subcellular structural localization analysis, the main subcategories (over 20%) for the SNI/Sham proteins were nuclear (33.33%) and extracellular (22.22%), while those for the EA/SNI proteins were cytoplasmic (37.74%) and nuclear (33.96%) (Figures 3(c) and 3(d)).

### 3.4. Functional Enrichment and Cluster Analysis of the SNI and EA Groups Using KEGG Pathway Analysis

**Figure 4:**
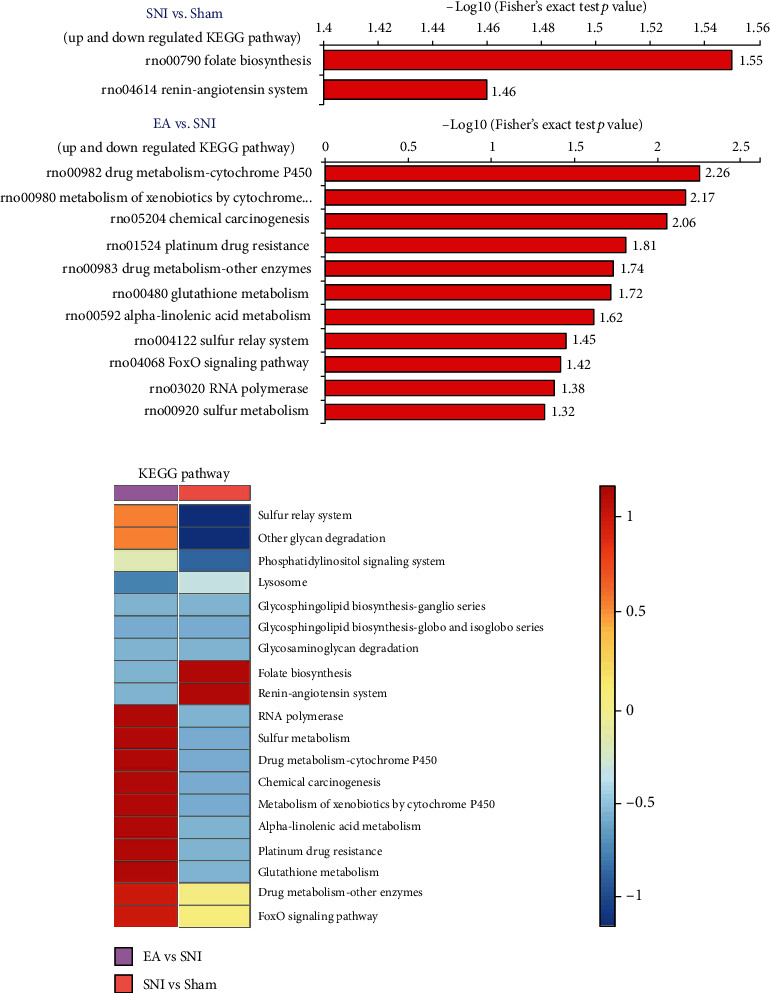
KEGG pathway analysis: (a) KEGG pathway enrichment analysis comparing differentially expressed proteins among the three groups; (b) KEGG pathway analysis of rats from the different groups.

To further explore which pathways were significantly affected by the differentially expressed proteins, the proteins were mapped to the KEGG database and their enrichment levels were calculated using the Fisher exact test *P* value [–log_10_(*P*)]. As shown in Figures 4(a) and 4(b), the KEGG pathway enrichment analysis demonstrated that M/S differentially expressed proteins were mainly enriched in renin–angiotensin system and folate biosynthesis pathways. In contrast, the E/M differentially expressed proteins were mainly enriched in the drug metabolism, cytochrome P450, and metabolism of xenobiotics by cytochrome P450 pathways.

## 4. Discussion

In our research, we found that 4 weeks of continuous EA treatment in rats significantly increased their mechanical pain threshold and improved cognitive deficits caused by spared nerve injury-induced neuropathic pain. This is the first time that hippocampal proteomic results have been published regarding spared nerve injury-induced neuropathic pain in rats treated with repeated EA stimulation. In total, we quantified 4,804 proteins using the TMT labeling proteomic method. Of these proteins, only 16 were upregulated, while 11 were downregulated during the development of neuropathic pain.

These differentially expressed proteins were mainly involved in the renin–angiotensin system and folate biosynthesis; for example, ACE2 and tryptophan hydroxylase-2 (TPH2), which were downregulated in the neuropathic pain model rats. Recently, some studies have shown that central ACE2 and TPH2 are involved in pain persistence and cognitive decline. ACE2 deficiency has been found to impair cognitive function, increase oxidative stress, and decrease BDNF levels in the hippocampus [29]. The number of ACE2-positive neurons was significantly decreased in diabetic neuropathic pain and is linked to the apoptosis of inhibitory neurons, such as GABAergic interneurons, in the spinal dorsal horn [30]. Tph2 shRNA expression in RVM neurons induced a significant downregulation of Tph2 in the RVM and 5-HT in spinal dorsal horn, attenuating nerve injury-induced allodynia [31].

According to the difference ratios in protein expression and the associated *P* values, we found that the most prominent upregulated or downregulated proteins, respectively, were the transmembrane protein 126A (TMEM126A) and the excitatory amino acid transporters (SLC1A2/EAAT2). Among the five known human EAAT subtypes, of the glial carriers, EAAT2 has the greatest impact on the clearance of glutamate released during neurotransmission. Spinal EAAT2 plays a critical role in both the induction and the maintenance of neuropathic pain. Sciatic nerve injury- (CCI-) induced pain caused an initial upregulation of EAAT2 on postoperative days 1–4, followed by a downregulation on days 7–14 [32]. Furthermore, upregulation and downregulation of EAAT2 are also apparent in the neuropathic pain [33]. With respect to the present study, the upregulation of hippocampal EAAT2 may be regarded as a central protective mechanism or a feedback regulation mechanism that prevents the adverse consequences of glutamate accumulation after nerve injury. Therefore, inhibition of EAAT2 expression using EA was able to alleviate the mechanical allodynia.

Further analysis of the proteomic data revealed spared nerve injury-induced downregulated proteins. These included TMEM126A, which was upregulated in the EA group, as confirmed by the ELISA/western blot results. TMEM126A encodes a mitochondrial protein found in higher eukaryotes that is important for providing mitochondrial energy. A series of studies have confirmed that auditory neuropathy is a key feature of TMEM126A-associated optic atrophy [34–37]. TMEM126A is a CD137 ligand-binding protein, which couples with the TLR4 signal transduction pathway in macrophages. It plays an important role in inflammatory immune regulation, increasing cell surface expression of proteins such as CD54, MHC II, CD40, and CD86. Cell-surface TMEM126A expression was found to increase after LPS induction in macrophages over 24 h, while CD137L expression decreased [22, 23]. Recent studies have shown that expression of the hippocampal microglia 1 surface marker CD86+ and microglial activation increase when neuropathic pain is induced by chronic peripheral nerve injury [8, 38]. In addition, expression levels of TLR4/NF-*κ*B signaling pathway-related proteins are upregulated in the hippocampus of neuropathic pain model mice [39].

To our surprise, we found that spared nerve injury-induced neuropathic pain downregulated TMEM126 expression, while TMEM126A was upregulated after EA treatment. This study is the first to demonstrate the role of TMEM126A in the central analgesic effect of EA. Combined with the results of the studies discussed above, we speculate that EA may play an analgesic role by regulating the expression of TMEM126A. This may inhibit the expression of hippocampal microglia and the microglial M1 marker, potentially reducing hippocampal neuroinflammation. In future experimental studies, we intend to construct a mouse model with altered expression of the TMEM126A gene or use TMEM126A shRNA to further analyze the hippocampal mechanism underlying EA-induced analgesia.

## 5. Conclusions

In conclusion, using the TMT labeling approach coupled with LC-MS/MS, we showed that spared nerve injury and EA stimulation drive significant changes in the levels of hippocampal proteins, especially those of inflammation-regulated proteins such as EAAT2 and TMEM126A. Our results may provide candidate protein biomarkers for the diagnosis and treatment of spared nerve injury.

## Figures and Tables

**Table 1:** Proteomic information and biological functions of TMEM126 and AEAAT2.

SNI vs. Sham most significant differential proteins	Transmembrane protein 126A	Excitatory amino acid transporters (SLC1A2/EAAT2)
Change ratio	0.64	4.681
*P* value	0.0016	0.000000212
Subcellular localization	Cytoplasm	Plasma membrane
KEGG orthology	K18157	K05613
Main role of current reports	Immune-inflammatory and tumor metastasis regulation [22–24]	Clearance of spinal glutamate released in pain states; restore hippocampus-dependent memory deficit [25–28]

**Table 2:** Information of common changed proteins in the three groups.

Protein accession	Protein description	SNI/Sham ratio (down-/upregulated)	*P* value	EA/SNI ratio (up-/downregulated)	*P* value	Gene name
Q5HZA9	Transmembrane protein 126A	0.64	0.0358	1.704	0.0016	Tmem126a
D3ZFR9	Retinol dehydrogenase	0.646	0.0433	1.817	0.0433	Rdh13
G3V9R0	LUC7-like	0.663	0.0033	1.688	0.0034	Luc7l
Q3KRE2	Methyltransferase-like 7A	1.23	0.0281	0.81	0.0334	MettL7A
Q5FVF3	ARF-binding protein 1	1.303	0.0012	0.746	0.02004	GGA1
Q6V7V2	Rhotekin	1.515	0.0241	0.727	0.0141	RTKN
D4A1U7	Round spermatid basic protein 1	1.996	0.0016	0.472	0.0023	Rsbn1
O08769	Cyclin-dependent kinase inhibitor	2.419	0.0002	0.445	0.00004	Cdkn1b

## Data Availability

The data used to support the findings of this study are available from the corresponding author upon request.
